# “Wild Years”: Rock Music, Problem Behaviors and Mental Well-being in Adolescence and Young Adulthood

**DOI:** 10.1007/s10964-021-01505-0

**Published:** 2021-10-11

**Authors:** Tom T. Bogt, William W. Hale, Andrik Becht

**Affiliations:** 1 Department of Interdisciplinary Social Science, Utrecht University, Padualaan 14, 3584 CH Utrecht, The Netherlands; 2 Research Center Adolescent Development, Utrecht University, Utrecht, The Netherlands

**Keywords:** Adolescence, Aggression, Depression, Drug use, Goth, Heavy metal, Music, Rock, Well-being

## Abstract

Adolescent preferences for non-mainstream types of rock music can be markers of adolescent problem behaviors, but no study has ever investigated whether this relationship continues into adulthood. In a six-wave study, 900 Dutch adolescents were followed from ages 12 to 21 (*Mage* T1 12.4, 51.1% girls), while reporting on depressive symptoms, mental well-being, aggression and drug use. A latent class growth analysis on their preferences for specific types of rock music revealed four fan groups. When these fan groups were compared to one another, in adolescence, the *all-out rock fans* displayed the highest peak in depressive symptoms and the lowest dip in well-being and the *rock/metal fans* reported the most aggression. And for both these groups, drug use increased at the onset of adulthood. *Pop fans* displayed a profile characterized by low depressive symptoms and aggression, and high in mental well-being. Finally, the *popular rock fans* held an in-between position between pop fans, on one side, and the all-out rock fans and rock/metal fans, on the other side. Thus, music preferences can be markers of problems, not only in adolescence but also in young adulthood. Still, music can enhance mood, helps to cope with problems, and peers in fan groups can provide support. This research focuses on the relationship between music and problem behaviors, specifically among members of the all-out rock fans and rock/metal fans, but many of these young people might have had more personal problems if they had not had their music and their fan-group peers.

## Introduction

Music is the soundtrack of adolescents’ journey into adulthood. In identifying with a specific body of songs, as well as with the creators of these songs and other fans, adolescents define and finetune their ideas about who they are, who they want to be, and with whom they want to socialize (North & Hargreaves, 1999). In adolescence music preferences “show who you are” and have been referred to as a “badge” (Frith, 1981; Rentfrow & Gosling, 2006). As lyrics and, more in general, personae and images of artists address a wealth of situations, cognitions and feelings, music is also a medium that can help in defining and tackling difficulties (Schäfer et al., 2013; Ter Bogt et al., 2016). But music preferences have also been connected to problem behaviors. Theories such as the *Music Marker Theory* (Ter Bogt et al., 2013) and the *Peer Group Mediation Model* (Slater & Henry, 2013) posit that young people facing problems may seek non-mainstream music as a way to cope with problems, but that within non-mainstream music scenes their problems may exacerbate, as they adopt the sometimes maladaptive attitudes and behaviors that are normative in these groups. Fans of different types of non-mainstream rock music have indeed been shown to be prone to school dropout, drug abuse, self-harm, and depression (for a review see: Lozon & Besimon, 2014). However, most of the studies in this field only cover the development of a specific (rock) music style or scene, and, mostly, rely on cross-sectional data. Of the few longitudinal studies on rock music and problem behaviors, none have followed fans into adulthood, to examine whether their problems are specific for adolescence or last into adulthood. Therefore, this study aims at identifying several distinct types of mainstream and non-mainstream rock fans, and follow them across adolescence, to explore heterogeneity in development of problem behaviors and mental well-being. It further aims to test whether adolescent problem behaviors dissipate when these fans find a more definite identity and place in life, or whether fans’ problems carry into their adulthood.

### Rock Music and Its Audience: A Brief History

Music labeled as “rock” encompasses a range of different styles. The history of rock music covers more than sixty years and is beyond the scope of this article. However, a few basics should be discussed in order to identify the various subtypes of rock music and their fans which are the focus of this research.

It is difficult to distill definite musical essentials for rock, but during the sixties and seventies of the last century it became synonymous with electronically amplified music, with a strong drum beat, and often, but not always, loud guitars and vocals. “Rock” cannot be fully distinguished from “pop” in musical terms, but these notions generally distinguish more “serious”, album-based rock music for (young) adults versus single-based “top of the pops” music for teenagers (Frith, 1981; 2020; Gillet, 1996). Another relevant distinction that partly overlaps the pop-rock division is mainstream versus non-mainstream. Mainstream music is often upbeat, melodic music in radio-friendly formats, directed at a large audience of listeners (Dowd, 2013). Pop music is by definition mainstream music. Melodic rock songs can be mainstream, but the brash, louder rock variants are often qualified as non-mainstream. Renfrow et al. (2011; 2012) indicated that “intense” (rock) music is experienced by listeners as animated and strong, as dense, distorted, and loud. By the mid and late sixties, bands such as The Who, Cream, Led Zeppelin, and the Jimi Hendrix Experience were called “rock” bands. In the last three decades of the past century, rock, both in mainstream and non-mainstream varieties, became a dominant popular music genre in the single and album charts, in addition to pop and soul music (Gillet,^1996^).

In the seventies and eighties an important subgenre appeared: heavy metal. Heavy metal artists criticized the softer, popular types of rock and veered back to what they believed was the core of the genre: extremely loud vocals, guitars and drums (Christe, 2003; Garofalo & Waksman, 2017). In the nineties and the first decade of the new century, two new popular subgenres developed in the spectrum of rock music: goth and emo rock. In the U.S., goth gained popularity (and notoriety) through the music of Marilyn Manson. With highly provocative lyrics, video clips and looks, “Anti-Christ Superstar” Marilyn Manson became one of the most successful bands of the late nineties and early new century (Wright, 2000). Emo is another non-mainstream rock genre and subculture originating from seventies punk and new wave, thematizing feelings of out-of-placeness and teenage angst (Greenwald, 2003). Not all bands in the off-mainstream rock scene were as bleak, nihilistic, violent and loud as Marilyn Manson or, for example, Cannibal Corpse (sic). The goth, emo and metal scenes produced more melodic, “symphonic” bands such as Within Temptation and Nightwish, that incorporated elements of classical music in their musical vocabulary and consolidated a more female audience.

Rock has evolved into a myriad of styles and substyles. The more melodic and mainstream forms reach a broad audience across socio-economic and gender groups. The non-mainstream subgenres are adored by a smaller, but still substantial, group of fans. While the vast majority of rock artists are male, newer styles such as goth or symphonic rock, involve more female musicians and singers. In terms of other demographic characteristics, the ethnic/racial composition of the group of rock musicians and their audiences is overwhelmingly white, at least in the U.S. and Europe.

### The Relationship between Music Preference and Adolescent Problem Behaviors

Music can address problems and help young people cope with difficulties. The existence of a relationship between music preferences and adolescent problems may, therefore, not come as a surprise. Particularly non-mainstream types of music –heavy metal, goth, emo, ganstarap– have been framed to directly cause problems, but the relationship between music listening and problems is far more intricate (Ter Bogt et al., 2013). Adolescents with emotional and behavioral problems –now to be referred to as simply problem behaviors– such as those who are bullied and feel out of place, may seek music and youth subcultures that address and reflect these experiences. Still, the immersion in these youth subcultures may also instigate or worsen problems. The relation between music and youth subcultures, on the one hand, and problem behaviors, on the other hand, might be the result of processes of both selection and influence (Young et al., 2006). Several authors have tried to describe and theorize on these processes in more detail.

In his *Theory of Media Delinquency*, Roe (1992; 1995) argues that the trajectory of individuals and groups within the social status hierarchy results in a specific segmentation of music audiences. Students that do well in school and anticipate high status refrain from socially disvalued media such as violent videos and extreme types of rock music; they demonstrate a preference for culturally acceptable music genres such as classical music, blues, jazz or pop. Conversely, students dissatisfied with school and anticipating occupational problems or failure tend to identify with a non-mainstream genre such as heavy metal music.

In their *Peer Group Mediation Model*, Slater and Henry (2013) propose that music and music videos are directly relevant for adolescent drug use, as adolescents may imitate this behavior. But, in addition to social-cognitive processes, another mechanism is important. Music and music videos provide early adolescents with social identities that precede and encourage involvement with peer groups that embrace such identities. Once involved with such peer groups, adolescents will not only adopt or finetune their attitudes and behaviors in the direction of peer norms, but they will also deepen their involvement with media types that drove them in the direction of their peer crowd. Hence, media and peers are part of a “reinforcing spiral” (Slater, 2007).

Ter Bogt et al. (2013) proposed a highly similar model in order to explain the relationship between music preferences and adolescent problems. *Music Marker Theory* assumes that the modelling of attitudes and behaviors in music media may lead to the adoption of these attitudes and behaviors among their fans. But the authors also introduce a socialization mechanism. Early adolescents, on average, already know which music they prefer. Through music, they are drawn to specific crowds, varying in problem behaviors. Peers explicitly or implicitly demand compliance to social group norms, thus stimulating the acquisition or reinforcement of norms, attitudes and behaviors consistent with those of the group. In groups characterized by more frequent internalizing or externalizing behaviors, and acceptance of these behaviors as normal, adolescent problem behavior is expected to occur more frequently and escalate more quickly (Franken et al., 2017). Music preferences function as an early marker of concurrent and later problems, working through peer group socialization.

Thus, the literature provides evidence for three social-cognitive, selection and socialization processes relevant for the relationship between adolescent music preferences and problem behaviors. First, adolescents may model their behaviors and attitudes on their selected music media. Secondly, an adolescent’s social position and pre-existing psychosocial problems can affect his/her choice of music. And finally, music preferences push adolescents in the direction of music scenes that are either more or less characterized by problems. Problem behaviors can be exacerbated in groups in which problem behaviors such as juvenile delinquency, drug use and misuse, depressive feelings, is the social norm, or at least tolerated.

### Empirical Studies on Rock Music, Problem Behaviors, and Mental Well-being

Research shows that fans of popular forms of rock music do not stand out as being problematic, though it must be noted that Dutch studies found more aggression and delinquency among those preferring rock compared to their peers that preferred pop (Mulder et al., 2007; Ter Bogt et al., 2013). However, this relations was qualified as weak, and compared to pop fans, rock fan did not show elevated the levels of internalizing problem behaviors and drug use.

A more problematic profile may be present among fans of heavier rock music. Deena Weinstein’s *Heavy Metal: A Cultural Sociology* (1991) and Jeffrey Arnett’s *Metalheads: Heavy Metal Music and Adolescent Alienation* (1996) are two defining studies of metal fans. Obviously, many metal fans are attracted to this music for its esthetic qualities, but a part of the audience is also attracted to metal as it reflects their position in life. As Arnett’s title suggests, many metal fans are alienated from institutions such as family, school, church, workplace, and from society at large. They are disproportionally the product of broken homes and at odds with their parents, teachers, bosses and other authorities. Heavy metal music lyrics thematize the fact that life can be miserable and the world a dark and hostile place. In contrast, the heavy metal scene feels as a safe haven, a social sphere to retreat to with likeminded peers. Weinstein discusses the marginal position of many metal fans in similar terms but adds an important positive qualification. Metal fans identify as “proud pariahs”, alienated indeed, but happy with and proud of the company of kindred spirits in a crowd that feels warm and inviting. Both authors depict metal concerts as a gathering of the tribe where social isolation, failure, anger, and worries are forgotten and transcended, and fans feel togetherness in a unique, uplifting socio-musical ritual.

Although, heavy metal and other non-mainstream music may represent a valuable resource for young people (Baker & Brown, 2016; Sharman & Dingle, 2015), these fan groups consistently show more behavioral problems than, for example, their pop music-oriented peers. Preferences for metal music are related to reckless behavior, aggression, drunk driving, drug abuse, delinquency, school dropout, depression and suicide ideation (Arnett, 1991; Hughes et al., 2018; Lacourse et al., 2001; Martin et al., 1993; Roe, 1992; Scheel & Westeveld, 1999; Selfhout et al., 2008; Tanner et al., 2008; Ter Bogt et al., 2013).

Scottish fans of goth rock are more inclined to self-harm and attempted suicide than those not identifying with this subculture (Young et al., 2006). A UK study found that liking goth at age 15 materialized as a powerful predictor for depression and self-harm at age 18. (Bowes et al., 2015). A Dutch study found that, for both girls and boys, goth music preferences emerged as an early marker of dormant or developing depressive symptoms (Ter Bogt et al., 2021). All these studies included strong sets of confounders that did not render goth’s effects insignificant. In a small scale, qualitative study, a relationship was reported between belonging to the emo rock subculture and high tolerance towards self-harm, suicide ideation and suicidal behaviors (Trnka et al., 2018). These results prompted all these researchers to conclude that members of the goth and emo rock scene are vulnerable young people.

The literature shows that many rock fans simply like the music because of its esthetic qualities, but a preference for heavy metal and other forms of non-mainstream rock may indicate a range of adolescent internalizing and externalizing problems. Male, white, working-class adolescents in distress made metal their music of choice in the eighties and nineties, as the music reflected their social position and problems, and the metal community offered a welcoming and soothing social environment. In recent times, the same holds true for a, in terms of gender and socio-economic status, more diverse group of fans of goth, emo and other non-mainstream rock.

However, there are some important limitations in the previous studies of the development of adolescent problem behaviors in rock music fans. Most studies have concentrated on only one type of music or a specific subgroup of rock fans, and the majority is cross-sectional. In the longitudinal studies on this topic, the focus lies often on only one subgroup of rock music fans. And in the few longitudinal studies that include several types of rock music fans, the statistical analysis is *variable centered* instead of *person centered*, implying that music is modeled as *factors* in multivariate analyses, and not as *groups* of fans for these analyses. However, the aforementioned theoretic models on the relationship between rock music and adolescent problem behaviors suggest that fan *groups* are a key element in the mechanism through which music preferences translate into problem behaviors. Therefore, a wide range of rock fan groups should be examined when exploring the relation between rock music types and problem behaviors. Furthermore, though it has now firmly been established that associations between preferring non-mainstream types of music and adolescent problem behaviors exist, it has never been investigated whether these problems remain over time; whether they are “adolescent limited” or “adolescent onset” (Moffitt et al., 2002; Odgers et al., 2008). It should be explored whether being part of an alienated youth culture or non-mainstream music scene may mark not only concurrent problems in adolescence but also future problems in early adulthood.

## Current Study

The literature lacks a person centered, longitudinal study following different rock fans across adolescence into adulthood, assessing their music preferences in relation to internalizing and externalizing problem behaviors, and well-being. Therefore, this study aims, first, at identifying the existence and development of various types of rock fan groups, including mainstream rock types, and non-mainstream types such as goth and heavy metal, or maybe even other fan groups with mixed prefrences. Second, this study aims to explore heterogeneity in fans’ problem behaviors (specifically, depressive symptoms, aggression, and drug use) and mental well-being across adolescence. Third, this study will examine if fans’ problem behaviors are best described as either adolescent limited problem behavior or remain present in adulthood. Based on the review of the literature it was hypothesized that at least three different fan groups within the rock spectrum can be discerned: fans liking popular rock, but not non-mainstream genres; fans preferring non-mainstream rock only; fans liking all types of rock music (Hypothesis 1). It was further assumed that fans liking popular rock will display less depressive symptoms, aggression and drug use, and more mental well-being, compared to fans more inclined towards non-mainstream rock (Hypothesis 2). Whether the problems and lower level of mental well-being of the last group disappear in young adulthood is open to question, but it is safe to assume that alienated adolescents in non-mainstream scenes may find it more difficult to integrate in adult life, and, thus, may remain more often problem ridden than mainstream music-oriented adolescents making a fluent transition into adulthood and adult responsibilities (Hypothesis 3). It should be stressed that, in line with the aforementioned theories, music is not identified and modelled as a causal agent; music preferences are conceptualized as markers of concurrent and future problem behaviors.

## Method

### Participants

Participants were 900 adolescents (*Mage* T1: 12.4 years, range 10–13 years, 51.1% girls) who participated in the early adolescent cohort of the CONflict And Management Of RElationships study (CONAMORE) (Meeus et al., 2004). CONAMORE is a prospective, longitudinal study that examines the relationships of Dutch adolescents with parents and peers as well as their emotional states. In the current study, data were used from six waves with a 1-year interval between waves 1 to 5, and a 5-year interval between wave 5 and 6.

### Procedure

Participants came from various high schools in the Dutch province of Utrecht. Parents and students received a letter in which the aims of the study were described, and information was given about the option of not participating. Participating students were required to provide written informed consent. Less than 1% decided not to participate. Participants completed a series of questionnaires in their classroom after school hours. Research assistants, who attended the administration, provided verbal instructions about filling out the questionnaires in addition to the written instructions printed above each questionnaire. Students were explicitly guaranteed confidentiality of their answers to the questionnaires. For students who were absent on the day of testing, a second assessment time was organized. Students who were absent on both days of testing were not assessed. At each of the 5 yearly waves, respondents received 10 euros. Five years later, when these students were young adults, they were again approached (mean age at T6: 21.3 years). The CONAMORE study was approved by the Ethical Review board of the Faculty of Social Sciences of Utrecht University.

Sample attrition across six waves was relatively low, with 78% of the adolescents who participated in the first wave still participating in the sixth wave. Thus, 22% of the participants dropped out over the course of the study. Next, we conducted additional analyses to test whether the drop-out group differed on any of the study variables compared to the participants. Results from a MANOVA revealed that adolescents who dropped out were slightly older 12.58 years vs. 12.32 years at W1, *F*(1, 133) = 5.84, *p* = 0.017, partial *η*^*2*^ = 0.04, and had slightly higher levels of depressive symptoms (1.25 vs 1.11, *F*(1, 133) = 10.41, *p* = 0.002, partial *η*^*2*^ = 0.07) compared to adolescents who participated both at wave 1 and wave 6. Participants did not differ on music preferences, aggression, drug use or mental well-being scores at wave 1 (all *p*s > 0.053). Boys were more likely to drop out over the course of the study compared to girls, *χ*^*2*^(1) = 21.60, *p* < 001, *φ* = 0.16. In all our subsequent analyses we included all participants with and without missing data. Missing data were handled in Mplus 8.4 using full information maximum likelihood (FIML).

### Measures

#### Demographic variables

At wave 1, respondents reported on their gender, age, and current level of education (High school vs. vocational training).

#### Music preferences

Adolescents’ music preferences were assessed by means of the Music Preference Questionnaire (MPQ) (Sikkema, 1999). The MPQ consists of a list of 13 established categories of music, including rock, heavy metal and goth music. Subjects were asked to indicate on five-point Likert scales “the extent to which they liked” each of the genres listed (from 1 = *do not like at all* to 5 = *like very much*). Option 6 indicated *do not know this type of music*. These scores were treated as missing values that were estimated in the analyses.

#### Depressive symptoms

The Children’s Depression Inventory (CDI) is a widely used self-report questionnaire of depressive symptomology in children and adolescents aged 8–18 years (Timbremont & Braet, 2002). The questionnaire is composed of 27 items that review various depressive symptoms categories such as mood, vegetative, cognitive, and psychomotor disturbances. The questionnaire is scored on a three-point scale (1 = *not true*, 2 = *a bit true* and 3 = *very true*). The total scores on the questionnaire can range from 27 to 81, with higher scores being reflective of greater depression. The CDI has strong internal consistency and validity in nonclinical populations (Saylor et al., 1984). Cronbach’s alpha for depressive symptoms ranged between 0.90 and 0.93 across waves 1‒6.

#### Mental well-being

Mental well-being was measured by means of the Cantril ladder (Cantril, 1965). This measures the feeling of general well-being and happiness. Respondents were asked to indicate on a ten-point scale how they generally feel (from 1 = *very bad* to 10 = *very well*).

#### Aggression

Aggression was measured by a self-report questionnaire, originally developed by Björkqvist et al. (1992). In the present study, only the subscale for aggression was used. This subscale consists of 17 items. Examples of these items are: When I’m mad at a classmate, I ‘call the other names’, ‘hit or kick’ and ‘curse’. The items were scored on a 4-point scale, ranging from 1 = *never*, 2 = *sometimes*, 3 = *often* to 4 = *very often*. In this study Cronbach’s alpha for aggression ranged between 0.85 and 0.93 across waves 1‒6.

#### Drug use

Two items in the Adolescent Criminal Behavior questionnaire (Baerveldt et al., 2003) measured adolescents’ cannabis use and use of other drugs than cannabis in the last 12 months. The items were also scored on the 4-point scale, ranging from 1 = *never* to 4 = *four times or more*.

### Statistical Analyses

First, it was examined whether different rock fan subgroups existed, based on their developmental shape in rock, heavy metal, and goth music preferences. To this end, a multivariate latent class growth analyses (LCGA; Jung & Wickrama, 2008) was conducted on the study’s data from all of the six measurement waves in *Mplus* 8.4 (Muthén & Muthén, 2017). To determine the number of latent classes that had the best fit to the data, the Bayesian Information Criterion (BIC; Schwarz, 1978) and the Lo-Mendell-Rubin adjusted Likelihood Ratio Test (aLRT; Lo et al., 2001) were used. A lower BIC value indicates a better fitting model. A significant aLRT indicates that a model with k classes fits better than a model with one class less (i.e., k - 1 classes). In addition, entropy, a measure of qualification certainty, should be acceptable (i.e., 0.75 or higher; Reinecke, 2006), and every class needs to cover at least 5% of the sample for meaningful interpretation and subsequent analyses (Muthén & Muthén, 2000). Subsequently, potential gender differences in the class distribution were explored.

Secondly, the development of problem behaviors (i.e., depressive symptoms, aggression, and drug use) and mental well-being were modelled across adolescence. To this end, four univariate latent growth curve models (LGMs) were conducted for each measure. From these LGMs, the intercept and slope factor scores for each individual were saved. Next, intercept and slope difference between the rock fan subgroups were tested. To account for classification error and keep the class distribution similar to the earlier LCGA class solution, a three step BCH approach was used (Asparouhov & Muthén,^ 2014^).

Finally, it was examined whether differences in depressive symptoms, aggression, drug use and mental well-being persisted from adolescence into young adulthood. To this end, a three step BCH approach was used to examine mean level differences in problem behaviors and well-being at T6 between rock fan subgroups.

Some of the scores on the study variables were non-normally distributed. Rock music had a relatively normal distribution, but scores on the heavy metal, goth, depressive symptoms, drug use and aggression variables were all positively skewed. Scores of mental well-being were negatively skewed. Histograms with the distribution of scores for all study variables across all waves are presented in online supplementary material S1. To account for the non-normal distributions of data, all models were estimated with the robust MLR procedure (Muthén & Muthén, 2017).

## Results

### Identification of Rock Fan Groups and Trajectories

**Fig. 1:**
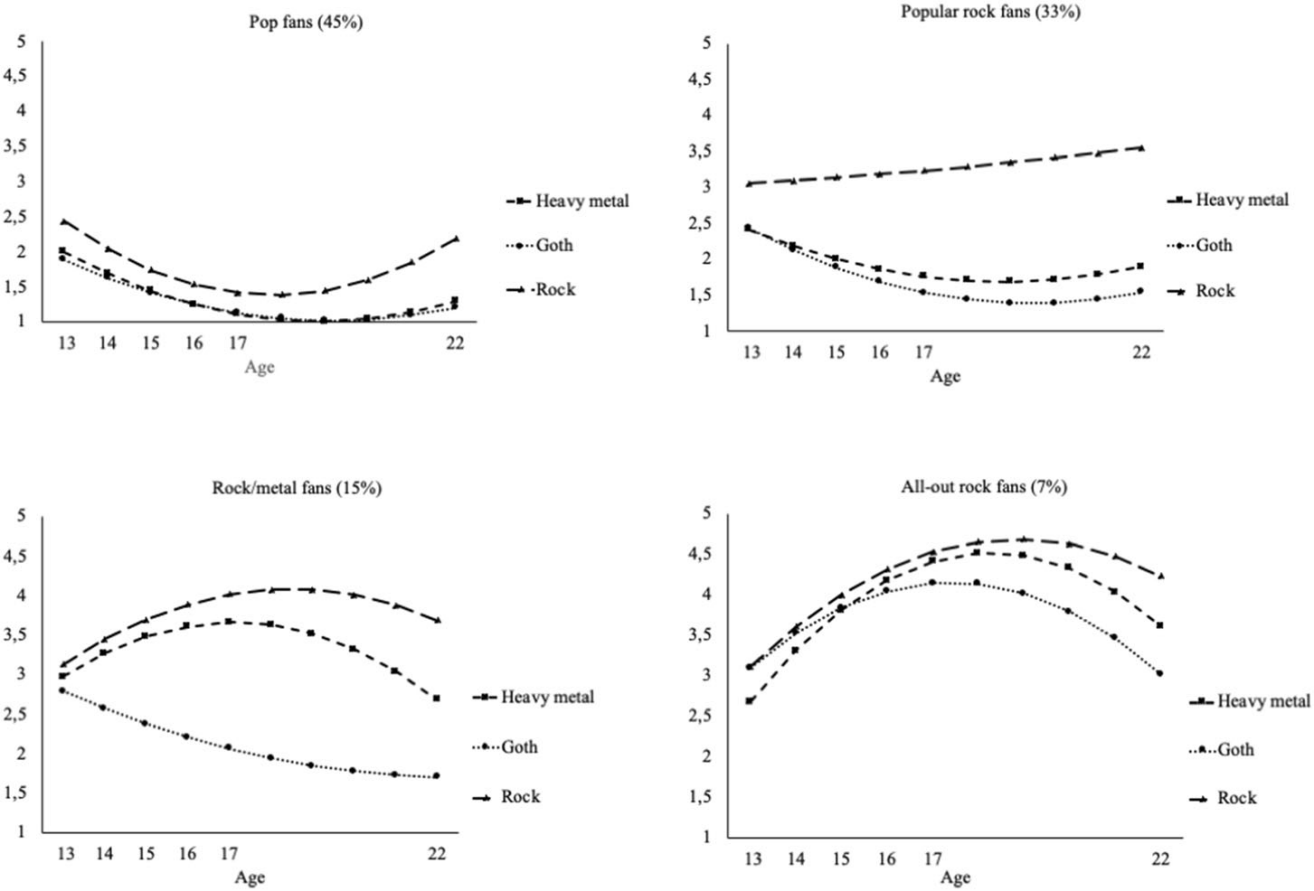
Final 4-class solution latent class growth curve analyses with estimated trajectories for rock, heavy metal, and goth music preferences across adolescence into young adulthood. Note. Range of music preferences scores depicted on the vertical axis: 1 (do not like at all) to 5 (like very much)

The first aim and hypothesis regarded the identification of rock fan groups. Prior univariate growth models revealed non-linear developmental trajectories of rock, heavy metal and goth music preferences (Ter Bogt et al., 2013). Therefore, we specified a multivariate quadratic LCGA for identifying different fan groups. Results revealed that a 4-class solution best fitted the data (BIC = 40078.11, aLRT *p* < 0.001, entropy 0.90). The BIC of this 4-class solution was lower than in the 1 through 3-class solutions, and a significant aLRT indicated that the 4-class solution provided a better fit with the data compared to a 3-class solution. While the BIC of the 5-class solution (BIC = 39792.04) was lower than the BIC of the 4-class solution (BIC = 40078.11), the aLRT of the 5-class solution was not significant (*p* = 0.062) and entropy (0.88) lower than for the 4-class solution (entropy 0.90). Therefore the 4-class solution was kept as the final model. Fit indices of all tested 1 through 5 class solutions are presented in online supplementary material Table S1. The estimated trajectories of the final 4-class solution are depicted in Fig. 1, and the exact parameter estimates of the intercept and slope factors of the 4 different classes can be found in Table 1.

**Table 1:** Parameter estimates of intercept and slope factors of rock subgenres across rock fans subgroups

Music Genres	Rock Fans Subgroups							
	Pop Fans (45%)		Popular Rock Fans (33%)		Rock/Metal Fans (15%)		All-Out Rock Fans (7%)	
	*M*	SE	*M*	SE	*M*	SE	*M*	SE
Rock								
Intercept	2.45***_a_	0.08	3.06***_b_	0.10	3.31***_b_	0.12	3.11***_b_	0.17
Linear Slope	−0.44***_a_	0.04	0.04_b_	0.06	0.35***_c_	0.07	0.54***_c_	0.07
Quadratic Slope	0.05***_a_	0.00	0.00_b_	0.01	−0.03***_c_	0.01	−0.05***_c_	0.01
Heavy Metal								
Intercept	2.01***_a_	0.07	2.41***_b_	0.12	2.97***_c_	0.17	2.77***_bc_	0.22
Linear Slope	−0.34***_a_	0.03	−0.25***_a_	0.05	0.34**_b_	0.11	0.70***_c_	0.10
Quadratic Slope	0.03*** _a_	0.00	0.02*** _a_	0.01	−0.04***_b_	0.01	−0.07***_c_	0.01
Goth								
Intercept	1.89***_a_	0.07	2.44***_b_	0.11	2.79***_bc_	0.16	3.10***_c_	0.20
Linear Slope	−0.28***_a_	0.03	−0.32***_a_	0.05	−0.23* _a_	0.09	0.48***_b_	0.09
Quadratic Slope	0.02 ***_a_	0.00	0.03***_a_	0.00	0.01 _a_	0.01	−0.05***_b_	0.01

Means with the same subscript do not differ significantly from one another. Note that the subscripts apply to each growth function in each rock subgenre (e.g., differences between mean intercepts of rock across the four classes)

*p < 0.05, **p < 0.01, ***p < 0.001

Individuals in the first trajectory class (45%) scored lowest on all three rock subgenres, especially on metal and goth. Additional analysis of the preferences of this group showed that they particularly liked the most popular type of pop music, that is, music represented in the pop charts. In order to not address this group with a negative identity –“rock haters”‒ they were characterized by a positive music identification. Therefore, this group of adolescents was labeled *pop fans*. The second trajectory class (33%) included individuals with a relatively high preference for rock but dislike of heavy metal and goth. Hence, this class was labeled *popular rock fans*. The third class (15%) included individuals that liked rock and heavy metal, but disliked goth music. Therefore, this class was labeled the *rock/metal fans* class. Finally, the fourth class (7%) included individuals that liked all rock subgenres and were labeled the *all-out rock fans*. This 4-class solution can also be interpreted as dividing mainstream fans in the pop and popular rock groups from non-mainstream fans in the rock/metal and all-out rock groups.

### Gender Differences in Rock Fan Groups

Chi-square analysis revealed significant sex difference in the distribution of boys and girls across rock fans subgroups, *χ*^2^(3) = 19.86, *p* < .001, *φ* = 0.149. Girls were more likely to be present in the all-out rock trajectory (*n* = 39 girls vs. *n* = 22 boys), and in the pop fans trajectory (*n* = 220 girls vs. *n* = 193 boys). Boys were more likely to follow the rock/metal fans trajectory (*n* = 45 girls vs. *n* = 87 boys). The number of boys and girls in the popular rock fans group was equal (*n* = 147 boys, *n* = 147 girls).

### Developmental Trajectories of Problem Behaviors in Rock Fan Groups

Regarding the second aim, it was tested whether rock fans subgroups differed in their development of problem behaviors and well-being across adolescence into young adulthood. To this end, four univariate latent growth model (LGM) analyses were conducted ‒depressive symptoms, aggression, drug use and mental well-being‒ to establish average growth trajectories. For all measures, a quadratic growth model provided the best fit with the data, as indicated by CFI values ranging between 0.93 and 1.00, and RMSEA values between 0.02 and 0.06. Figure S1 shows the univariate growth trajectories for each behavior outcome.

**Fig. 2:**
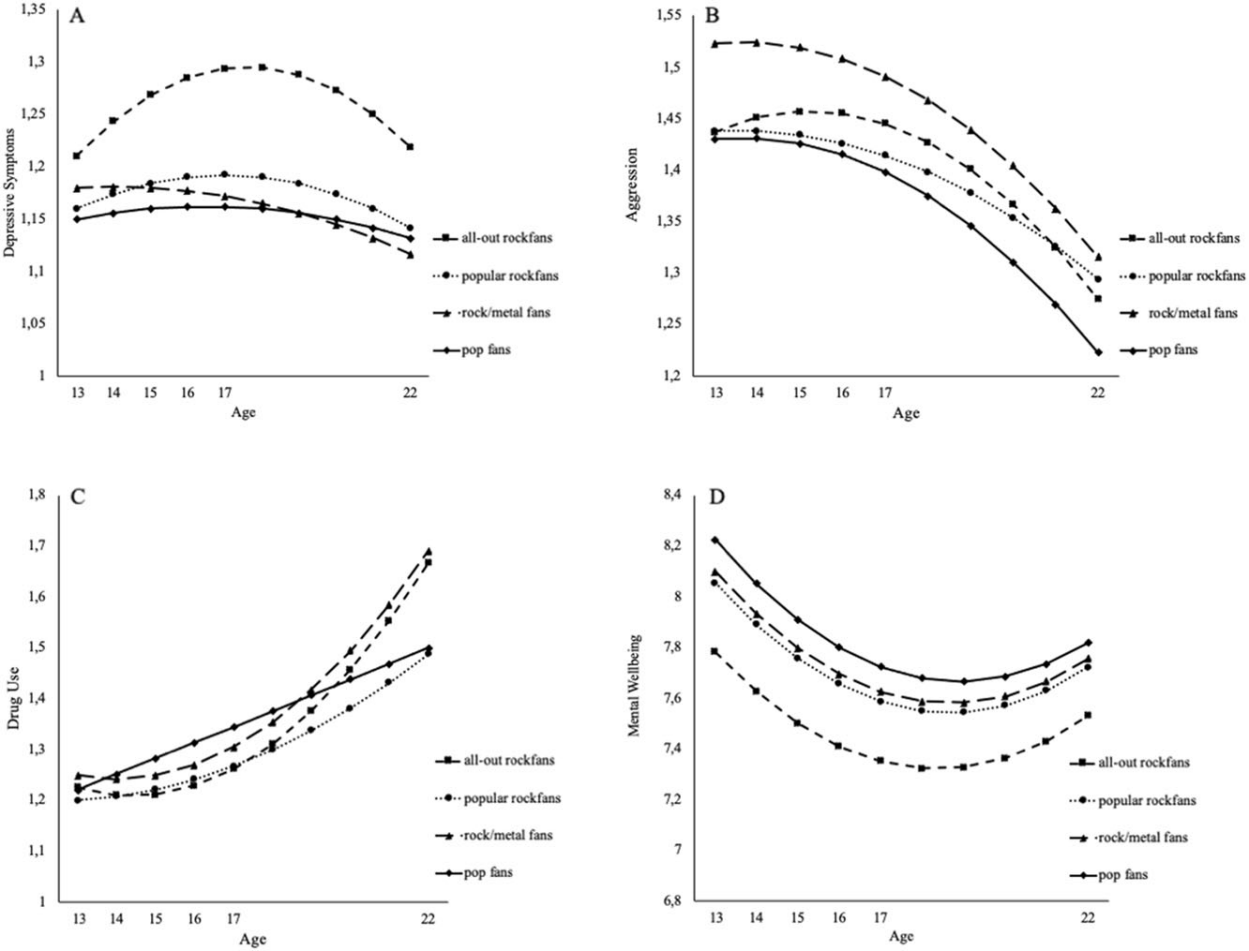
Differences in development of depressive symptoms (panel **A**), Aggression (panel **B**), Drug Use (panel **C**), and Mental Well-being (panel **D**) across rock fans subgroups. Note. Range of scores depicted on the vertical axis: Depressive symptoms 1–3, Aggression 1–4, Drug Use 1–4, and Mental Well-being 1–10

Next, intercept and slope differences were examined across different rock fans subgroups. Table 2 shows the parameter estimates and differences between rock fan subgroups in intercept and slopes for depressive symptoms, mental well-being, aggression, and drug use. Figure 2 depicts the developmental trajectories of problem behaviors across rock fans subgroups.

**Table 2:** Mean differences between rock fans subgroups in intercept and slopes of problem behaviors and well-being trajectories

Growth Factors	Pop Fans	Popular Rock Fans	Rock/Metal Fans	All-Out Rock Fans
Depressive				
Symptoms	1.15*_a_	1.16*_a_	1.18*_ab_	1.21*_b_
Intercept	0.01*_a_	0.02*_b_	0.00*_a_	0.04*_c_
Linear Slope	−0.001*_a_	−0.002*_b_	-0.001*_a_	−0.004*_c_
Quadratic Slope				
Mental Well-being				
Intercept	8.23*_a_	8.06*_b_	8.10*_ab_	7.78*_c_
Linear Slope	−0.19*_a_	−0.18*_b_	−0.18*_b_	−0.17*_c_
Quadratic Slope^1^	0.02*_a_	0.02*_a_	0.02*_a_	0.02*_a_
Aggression				
Intercept	1.43*_a_	1.44*_a_	1.52*_b_	1.44*_a_
Linear slope	0.004*_a_	0.02*_a_	0.004*_a_	0.02*_a_
Quadratic slope	−0.003*_ab_	−0.002*_a_	−0.003*_ab_	−0.004*_b_
Drug Use				
Intercept	1.22*_a_	1.20*_a_	1.25*_a_	1.23*_a_
Linear Slope	0.031*_b_	0.01*_a_	−0.01*_a_	−0.02*_a_
Quadratic Slope	0.000*_a_	0.003*_b_	0.007*_c_	0.008*_bc_

Means with the same subscript do not differ significantly from one another

^1^The quadratic latent growth model for mental well-being only converged when fixing the variance of the quadratic slope factor to zero. Hence, participants did not differ from each other in their quadratic slope value

*p < 0.05

#### Depressive symptoms

Pop fans and popular rock fans showed a significantly lower depressive symptoms intercept compared to all-out rock fans. No other intercept differences were found. Over time, all-out rock fans showed a peak in depressive symptoms around age 17 years, that resulted from a stronger linear increase and subsequent quadratic decrease in depressive symptoms, compared to pop fans and rock/metal fans. Although less pronounced than the all-out rock fans, the popular rock fans showed a stronger increase and subsequent decrease in depressive symptoms as well, compared to pop fans and rock/metal fans.

#### Mental well-being

All-out rock fans reported the lowest intercept of mental well-being compared to all other classes. Popular rock fans showed a lower intercept of well-being compared to pop fans. Moreover, both pop fans and all-out rock fans showed the steepest decrease in mental well-being compared to popular rock fans and rock/metal fans. The slopes of pop fans and all-out rock fans did not differ significantly from each other. However, because all-out rock fans started with a lower baseline/intercept of mental well-being the dip in mental well-being was most pronounced for the all-out rock fans compared to the other classes.

#### Aggression

Rock/metal fans reported a higher intercept of aggression compared to all other classes. Only one significant difference in development of aggression between rock fans subgroups was found; all-out rock fans showed a slightly steeper quadratic decline of aggression into young adulthood compared to popular rock fans.

#### Drug use

No intercept differences in drug use were found between rock fans subgroups. However, pop fans showed a significantly stronger linear increase in drug use compared to all other classes, that did not differ from each other. Rock/metal fans and all-out rock fans had a stronger positive quadratic slope compared to pop fans. As a result, both rock/metal fans and all-out rock fans showed a diverging developmental pattern with stronger increases in drug use into young adulthood, compared to pop fans and popular rock fans.

### Problem Behaviors in Young Adulthood

For the third aim, it was examined whether problem behaviors and a lack of mental well-being persisted from adolescence into young adulthood by comparing mean levels of these measures in young adulthood (at T6) across the four different types of rock fans and non-fans. Results revealed no significant differences at T6 for depressive symptoms and mental well-being. However, drug use and level of aggression differed between classes in young adulthood. Concerning drug use, at T6 all-out rock fans reported significantly higher drug use levels compared to pop fans (*p* = 0.032). Rock/metal fans reported significantly higher drug use levels compared to popular rock fans (*p* = 0.002) and pop fans (*p* < 0.001). Rock/metal fans also reported more aggression at T6 compared to pop fans (*p* = 0.039). Finally, popular rock fans reported more aggression compared to pop fans (*p* = 0.001).

### Gender Differences

Additional sensitivity analyses in MANOVAs with rock fans subgroups as independent variables, gender added as a covariate, and with intercepts, slopes and mean scores at T6 of problem behaviors and mental well-being as dependent variables, revealed virtually similar results.

## Discussion

Music is a highly relevant medium for adolescents and young adults. Music enhances mood, helps to develop an identity, but has also been related to adolescent problems. The literature on music and problem behaviors lacks longitudinal studies tracing a range of specific music fan groups across adolescence. This study focusses on the rock spectrum of music and uncovers different types of rock fans and non-fans, follows them from early adolescence into adulthood, and explores the development of depressive symptoms, aggression, drug use and mental well-being in these groups.

The fist aim was to investigate whether different fan groups can be identified based on preferences for not only rock music but also heavy metal and goth. It was hypothesized that at least three groups would be present: fans liking popular rock but not non-mainstream genres; fans preferring non-mainstream rock only; and fans liking all types of rock music. Results of the multivariate latent class growth curve analyses (LCGAs) largely confirm this hypothesis but also revealed a fourth group with specific music developmental patterns: adolescents disliking all kinds of rock music. Nearly half (45%) of the respondents can be characterized as pop fans, as they do not like any kind of rock music and prefer mainstream pop music. A second, large group consists of popular rock fans (33%), preferring (mainstream) rock but disliking non-mainstream genres such as goth and heavy metal. Third, rock/metal fans (15%) favor both rock and metal but are not particularly fond of goth. Last, a small group of all-out rock fans (7%) likes all three genres. Thus, rock emerges as a relevant music style discriminating between a large group of non-fans (45%) and an even larger group of three types of fans adding up to 55% of the sample. These results also indicate a difference between mainstream pop and popular rock fans on the one hand, and non-mainstream rock/metal and all-out rock fans on the other.

Music preferences seem to be most outspoken in late adolescence (see Fig. 1), with preferences and dislikes for specific genres peaking at this age. Pop fans dislike rock, particularly in late adolescence; popular rock fans show a stable and slightly increasing preference for rock but strongly dislike other rock genres in late adolescence, just like pop fans. Rock/metal fans show an increasing preference for both rock and heavy metal in early adolescence, peaking in late adolescence and decreasing thereafter. This pattern is the most explicit for the all-out rock fans, demonstrating the steepest increases in early to middle adolescence, highest peaks in late adolescence and decreases into young adulthood, when compared to other fans. Several authors have referred to music preferences as a “badge” that demonstrates who you are or want to be, signaling to peers with whom you want to socialize (Frith, 1981; North & Hargreaves, 1999; Rentfrow & Gosling, 2006; Selfhout et al., 2009). These results demonstrate that this badge function of music taste may be most present and functional in late adolescence and may lose some of its significance in young adulthood. Rock/metal and all-out rock fans, to a certain extent, move back to the mainstream by no longer really adoring the rock genres that are disliked with a vengeance by mainstream pop and rock fans. In other words, differences between adolescent fans are most significant in late adolescence, but music loses some of its discriminative power in young adulthood.

The second aim was to test whether fan groups differ in the prevalence and development of depressive symptoms, aggression, drug use, and mental well-being across adolescence and into young adulthood. The third aim regarded the question whether problems and lack of mental well-being are typical adolescent phenomena or whether they carry over into adulthood. Our results provide evidence that adolescents in the non-mainstream groups of all-out rock fans and rock/metal fans indeed show more problems compared to their peers, particularly when compared to the pop fans group. Our results corroborate the conclusions of earlier qualitative and cross-sectional studies that loud, rebellious music indicates adolescent problem behaviors (Arnett, 1996; Lozon & Besimon, 2014; Trnka et al., 2018; Weinstein, 1991). However, the approach in the current study further nuances the relationship between music and problem behaviors. For example, all-out rock fans differentiate themselves across adolescence with more internalizing problem behaviors (such as, depressive symptoms and a lower degree of mental well-being), while rock/metal fans more often have more externalizing problem behaviors (such as, aggression). Both these fan groups display rapidly increasing drug use in late adolescence and early adulthood. It is reassuring though, that in young adulthood the two non-mainstream fan groups no longer show more depressive symptoms and lower levels of mental well-being than their peers in other fan groups.

More specifically, in early adolescence all-out rock fans show higher depressive symptoms rates than pop fans and popular rock fans. All-out rock fans’ depressive symptoms increase more rapidly up to age 17 than in any groups, and decrease more rapidly thereafter, implying that a peak in depressive symptoms in late adolescence is typical for this group. This peak in depressive symptoms is reversed in a dip in mental well-being that is, again, more pronounced in this group than in other groups. The all-out rock fans do not stand out regarding aggression. On the contrary, their aggression fades away more rapidly compared to, for example, popular rock fans. All-out rock fans seem to continue their drug use into adulthood. In late adolescence drug use increases sharply and in young adulthood is higher than among pop fans. Considering the entire period from early adolescence into young adulthood, the findings tentatively suggest that all-out rock fans continue to show the highest risk for problem behaviors, albeit for different types of problem behaviors in adolescence versus young adulthood. It may be that they come to terms with their inner demons when reaching adulthood and finding a more definite identity, but elevated depressive symptoms seem to translate into more drug use, a process that should be researched further.

In early adolescence, rock-metal fans already report more aggression compared to all other subgroups. Their trajectory across adolescence does not divert from other groups, implying that they keep on showing the highest scores on aggressive behavior that decreases in late adolescence and young adulthood. Still, in young adulthood their aggression levels are higher than among pop fans. And similar to the all-out rock fans group, their increasing drug use carries over into adulthood. Rock/metal fans are not particularly different from their peers regarding depressive symptoms and mental well-being.

The healthiest developmental pattern is present in the pop fans group. Their depressive symptoms and aggression follow a U-shaped trajectory and an inversely U-shaped trajectory for mental well-being. Overall, they show less depressive symptoms and aggression that the non-mainstream rock groups, and their mental well-being is higher across adolescence into young adulthood. Their drug use increases more strongly in early and mid-adolescence, but in young adulthood it is lower than in the all-out and rock/metal groups.

The popular rock fans are an in-between group. In early adolescence they do not divert much from their peers in the pop fans group with low depressive symptoms, aggression and drug use, though their mental well-being is lower. Across adolescence they show a pronounced increase in depressive symptoms, and in young adulthood they report more aggression, but their drug use remains low.

In conclusion, for many young people adolescence is a period in which depressive symptoms, aggression and drug use increase and mental well-being decreases, but these developments are most pronounced in non-mainstream fan groups: the rock/metal fans (15%) and all-out rock fans (7%). Compared to a reference group of pop fans, all-out rock fans show the highest peak in depressive symptoms and lowest dip in mental well-being. Rock/metal fans differentiate themselves in terms aggression. Both rock/metal and all-out rock fans show a pattern of developmental divergence with strong increases in drug use in late adolescence and early adulthood compared to pop-fans/and popular rock fans. These findings highlight that adolescence/young adulthood is a problematic period for non-mainstream fans and being part of these fan scenes indicates more drug use or aggression in young adulthood. On the positive site: it is reassuring that in young adulthood no remaining differences were found between pop fans and non-mainstream fans in terms of depressive symptoms and mental well-being.

A small number of longitudinal studies have tracked non-mainstream fans in adolescence and found them to be vulnerable in terms of internalizing and externalizing problem behaviors (Bowes et al., 2015; Slater & Henry, 2013; Ter Bogt et al., 2013, 2021; Young et al., 2006, 2014). This study discovered that preferences for non-mainstream types of music may not only be a marker of problem behaviors in adolescence but also predict more problems in young adulthood. As such, these findings closely resemble Moffit, 1993 developmental principle that the rock/metal and all-out rock fans’ problems are not “adolescence limited” but “adolescent onset” (Moffit, 1993; Odgers et al., 2008). Most problematic remains their accelerated increase in drug use in young adulthood. Still, it is important to stress that during the transition to adulthood, for these vulnerable non-mainstream groups of rock fans, depressive symptoms and aggression decrease substantially, and mental well-being increases. The term “wild years” **–**the ups and downs in mood, the sensation seeking behaviors**–** may be most appropriate to describe their adolescence.

### Strengths and Limitations

This study is characterized by several strengths. First, it was the first to follow a variety of rock music fans from early adolescence into young adulthood. Second, it connected fan development trajectories with longitudinal trajectories of a range of problem behaviors and well-being across adolescence into early adulthood. Inevitably, there were limitations to this study as well. First, in focusing on fan groups this study draws on concepts developed in the *Peer Group Mediation Model* (Slater & Henry, 2013) and *Music Marker Theory* (Ter Bogt et al., 2013). Though a key assumption of these theories is corroborated **–**music preferences indicate, foreshadow, and mark problems**–** the analytic strategy did not involve the process of peer mediation itself. Second, differences in problem behaviors across subgroups were significant but small. Small effect sizes may function as a warning to not stereotype fans. The proportion of metal/rock and all-out rock fans showing problems is higher than among pop fans or popular rock fans, by no means all of them can be characterized as “problematic”. Third, rock is an umbrella term and, as this paper shows, differences within the broad rock fan community are relevant not only in terms of music preferences themselves, but also in relation to the outcomes that were studied. That said, future research should discriminate more finely between fans, particularly within the group that emerged as “rock haters” and was subsequently labeled as pop fans in this investigation. A more complex characterization based on a larger number of music preferences might result in the discrimination of “true” pop fans and, additionally, other groups that, for example, like two other types of highly popular music, hip hop and dance. Again, a yet finer distinction might uncover larger mainstream and smaller non-mainstream hip-hop and dance fan groups. Fourth, rock music’s audience is predominantly white. By focusing on rock as a basis for the differentiation in fan groups, the resulting fan groups may be biased in terms of ethnicity. As already suggested, future studies should include a broader range of mainstream and non-mainstream types of music **–**R&B, hip hop, techno trance, hardhouse, classic music**–** and trace the development of their fans. The resulting groups can be compared to pop fans, that, again, emerged as a large group of well-integrated and non-problematic youth. Fifth, rock music is a world-wide phenomenon. Most of the studies on music and problem stem from the U.S. or a selective set of countries in Europe: Sweden, UK, and The Netherlands. It is important to replicate studies across different contexts to explore whether the meaning and effects of mainstream and non-mainstream types of music are similar in different cultural settings. Sixth, defining qualitative studies on fans (Arnett, 1996; Weinstein, 1991) have shown that non-mainstream scenes may literally be a lifeline for some members. Both the music itself and the company of like-minded young people, often facing similar problems and feelings of alienation, provide comfort and a welcoming social environment. The longitudinal analyses in this study rely on sophisticated statistical tools but were insensitive in terms of modeling the fine mechanisms through which music preferences can translate into problem behaviors or, alternatively, buffer problems. And the data that were collected did not include measures of positive effects of music listening or fan group membership. Presently no longitudinal study has disentangled positive and negative effects of belonging to non-mainstream music fan scenes. Future studies should do so.

## Conclusion

Music is important to most young people and it is a benevolent force in their lives. Music improves mood, soothes when in trouble, energizes at parties and helps you find friends; it may be key to your own identity. But this study also uncovers that non-mainstream music preferences may indicate or foreshadow problems in adolescence and even in young adulthood. These results primarily point at negative consequences, but it should be recalled that many of these young people might have been in a more troublesome state if they had missed the music and their friends. Future studies should try to better extricate different consequences, both positive and negative, of being part of non-mainstream fan scenes.

## Supplementary Information

Online supplementary materials
