# Supplementary material for: “Wild Years”: Rock Music, Problem Behaviors and Mental Well-being in Adolescence and Young Adulthood
Source: J Youth Adolesc. 2021 Oct 11;50(12):2487–500. doi: 10.1007/s10964-021-01505-0 (PMC8580930; doi:10.1007/s10964-021-01505-0)
Supplement: Supplementary file 1 — Online supplementary materials [file 10964_2021_1505_MOESM1_ESM.docx]

**Online supplementary materials**

**Table S1.**

*Fit Indices of 1-5 Class LCGA Solutions*

| Number of Classes |  | BIC | aLRT | Entropy | Class % |
| --- | --- | --- | --- | --- | --- |
| 1 |  | 45726.366 |  |  | 100 |
| 2 |  | 41840.950 | p = .000 | 0.953 | 75, 25 |
| 3 |  | 40613.996 | p = .022 | 0.903 | 48, 35, 17 |
| **4** |  | **40078.060** | **p = .000** | **0.903** | **45, 33, 15, 7** |
| 5 |  | 39792.038 | p = .062 | 0.876 | 39, 24, 15, 15, 7 |

*Note.* LCGA = Latent Class Growth Analyses, BIC = Bayesian Information Criterion,

aLRT =adjusted Lo-Mendell-Ruben likelihood ratio Test.

In bold type: the final 4-class solution.

**Figure S1.**

*Univariate Latent Growth Curve Models for Depressive Symptoms (panel A), Aggression (panel B), Drug Use, (panel C), and Mental Well-being (panel D)*

*
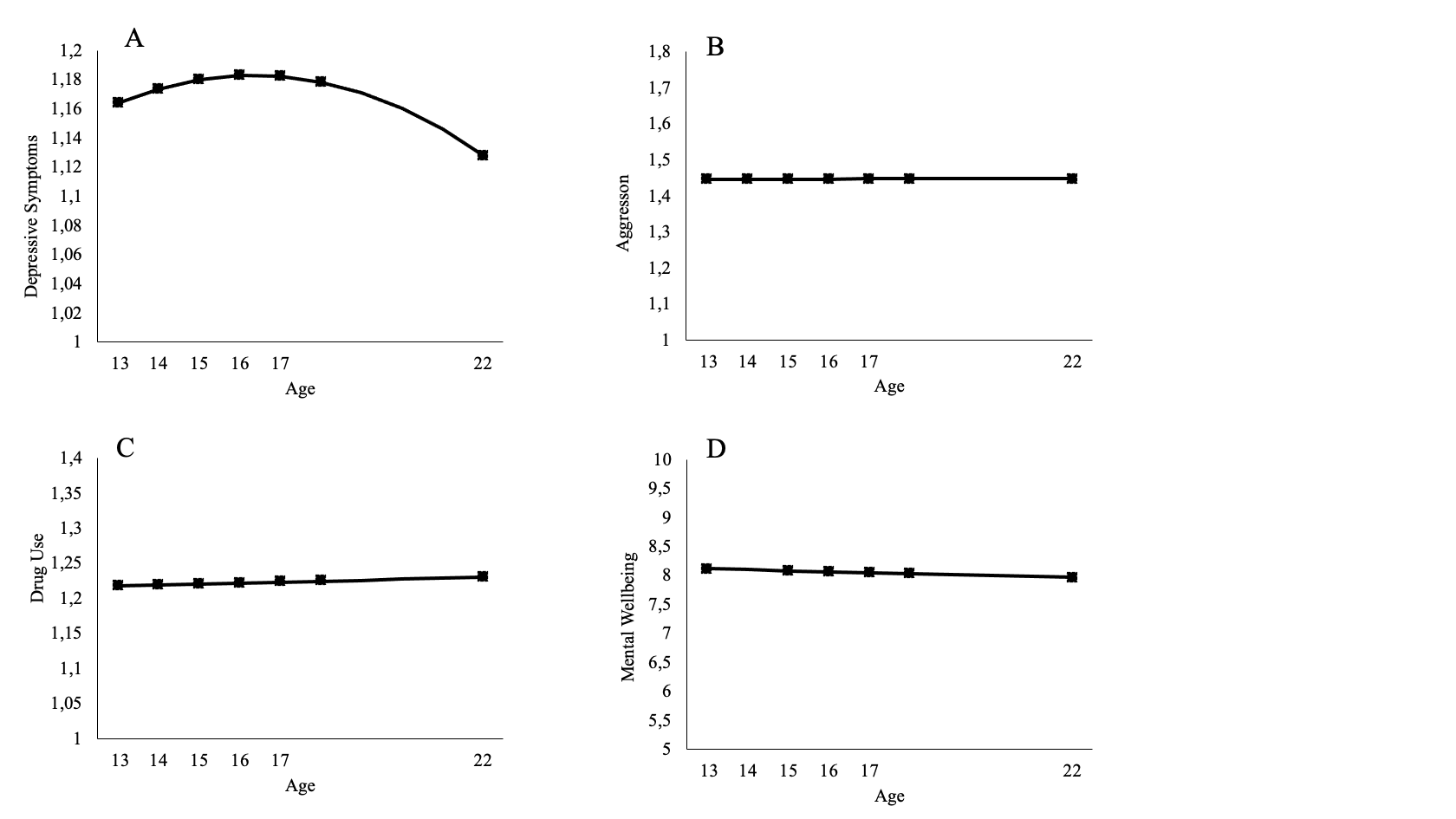
*

*Note.* Mental well-being was not assessed at wave 2 (14 years of age). Range of scores depicted on the vertical axis: Depressive symptoms 1–3, Aggression 1–4, Drug Use 1–4, Mental Well-being 1–10
